# Multiple mycoviruses identified in *Pestalotiopsis* spp. from Chinese bayberry

**DOI:** 10.1186/s12985-021-01513-3

**Published:** 2021-02-23

**Authors:** Fangyong Chen, Zhanxu Pu, Haizhi Ni, Yin Wang, Bangguo Yan

**Affiliations:** Citrus Research Institute of Zhejiang Province, Taizhou, 318026 China

**Keywords:** *Pestalotiopsis* spp., RNA-seq, Mycovirus

## Abstract

**Background:**

Chinese bayberry (*Myrica rubra*) is a subtropical fruit crop widely grown in southern China. Twig dieback is a disease of Chinese bayberry caused by *Pestalotiopsis* spp. and results in great economic losses to Chinese bayberry production. A virus survey was conducted in the population of *Pestalotiopsis* spp. infecting *M*. *rubra* in China. We explored the viral diversity in *Pestalotiopsis* spp., which may provide resources for further development as biocontrol agents of twig dieback.

**Methods:**

Strains of *Pestalotiopsis* spp. were isolated from diseased twigs of *M*. *rubra*, and cultured on potato dextrose agar for RNA extraction. The total RNA of each strain was extracted, mixed, and used for RNA sequencing. The resulting sequences were deduplicated, annotated, and then used for phylogenetic analysis.

**Results:**

Seven novel viruses were characterized from 59 isolates of *M*. *rubra* collected from 14 localities in China. Based on the phylogenetic analysis, these viruses were classified into five viral families/orders, *Botourmiaviridae*, *Mitoviridae*, *Partitiviridae*, *Tymovirales* and *Bunyavirales*, and one virus, Pestalotiopsis negative-stranded RNA virus 1, which likely belongs to a new viral family.

**Conclusions:**

Metatranscriptomics analysis showed the presence of various mycoviruses in *Pestalotiopsis* spp. isolated from *M*. *rubra* in China. The genomes of eight putative viruses were identified, seven of which were nearly full-length. Some of these viruses of *Pestalotiopsis* spp. may have the potential for the biological control of twig dieback of *M*. *rubra*.

**Supplementary Information:**

The online version contains supplementary material available at 10.1186/s12985-021-01513-3.

## Introduction

Chinese bayberry (*Myrica rubra*) is a subtropical fruit crop with great economic value that is widely grown in southern China. The fruits of *M*. *rubra* are not only eaten fresh but also can be processed into various products, such as juice, jam, sweets, and wine [1]. Recently, a disease causing twig dieback of *M*. *rubra* was found in all major Chinese *M*. *rubra* producing areas [2, 3]. The disease causes brown spots on the leaves and twigs of the diseased plants during the early stages of infection, and the infected trees experience leaf drop and may die within 1–4 years [2]. The disease spreads quickly and is becoming a serious threat to the development of *M*. *rubra*. Many species of *Pestalotiopsis*, like *Pestalotiopsis mangiferae*, *P*. *vismiae*, *P*. *microspora*, and *P*. *versicolor*, were determined to be the causal agents of twig dieback of *M*. *rubra* in China [2–4]. Besides being a plant pathogen, *Pestalotiopsis* spp. are also endophytes in many plant species, representing a huge and largely untapped resource of natural products [5].

Mycoviruses or fungal viruses refer to viruses parasitizing or inhabiting fungi and oomycetes. They are widely discovered in all major groups of fungi. The earliest record of mycoviruses was believed to be the discovery of virus-like particles in diseased *Agaricus bisporus* [6]. Due to the potential of using mycoviruses for the control of fungal diseases of plants [7] and the rapid development of sequencing technology, the presence of mycoviruses has been investigated in many different fungi, quickly increasing the number of known mycoviruses [8–12]. Mycoviruses are generally classified according to their hosts, genomic structure, and shape of virus particles, and most of them possess RNA genomes of double-stranded (ds) RNA (*Chrysoviridae*, *Totiviridae*, and *Paritiviridae*, among others), positive single-stranded (+ ss) RNA (*Alphaflexiviridae*, *Betaflexiviridae*, and *Narnaviridae*, among others), or negative single-stranded (-ss) RNA (*Mymonaviridae*). Nevertheless, many mycoviruses remain unclassified [13]. Besides RNA viruses, a few ssDNA viruses have been reported, such as *Sclerotinia sclerotiorum* [14] and *Fusarium graminearum* [15].

Next-generation sequencing (NGS) technology is a popular way to explore mycoviral diversity, including sequencing the total RNA that is depleted of ribosomal RNA or small RNAs [8–11]. Many novel viruses have been detected using NGS. For instance, 72 partial or complete genome segments, representing 66 previously undescribed mycoviruses, were identified in the metatranscriptomes of five widely distributed plant pathogenic fungi [10]. Similarly, 34 contigs, representing novel viruses, were identified in the virome of an Australian *S. sclerotiorum* population [12]. However, no viruses have been reported in the population of *Pestalotiopsis* spp. Therefore, in this study, we aimed to identify the viral population in *Pestalotiopsis* spp. infecting *M*. *rubra* in China by analyzing the total RNA with NGS technology.

## Materials and methods

### Isolates and growth conditions

In 2019, strains of *Pestalotiopsis* spp. were isolated from diseased twigs of *M*. *rubra* collected from 14 localities (Additional file 1: Table S1) in China, and then identified according to the method previously described [2, 3]. The stock cultures were maintained in potato dextrose agar (PDA) plates at 4 °C. Working cultures were established by transferring stock agar with mycelium onto PDA at 20 °C for 1 week.

### Total RNA extraction and purification

The total RNA of each strain was extracted from 0.1 g of fungal mycelium by using a Plant RNA Extraction Kit (Omega, America). The total RNA was stored at -80^˚^C until use. Approximately 2 μg of RNA were taken from each sample (59 strains) and then mixed. A mixed RNA sample (~ 120 μg) was sent to Novogene (Beijing, China) for RNA sequencing (RNA-seq).

### RNA sequencing and sequence analysis

Ribosomal RNA depletion (Ribo-Zero rRNA Removal Kit, Illumina, Inc.), library preparations (~ 1 μg RNA sample, TruSeq RNA Sample Preparation Kit, Illumina, Inc.) and Illumina sequencing (Illumina Novaseq 6000) were performed by Novogene (Beijing, China). The filtered data was spliced from scratch using Trinity software (version: 2.3.3), and the resulting sequences were then deduplicated with cd-hit (version: 4.6). Finally, Diamond software (version: 0.8.22) and the non-redundant protein database in the National Center for Biotechnology Information (NCBI) (https://www.ncbi.nlm.nih.gov/) were used for BLASTx annotation, and the viral sequences were selected. Pairwise sequence comparisons were carried out for both botourmiavirus and mitovirus related sequences, using Sequence Demarcation Tool (SDT) v.1.0 with the MUSCLE alignment option [16].

### Confirmation of viral-like contigs

To confirm the presence of viral-like contigs in the tested strains, the presence of each contig was determined by using RT-PCR with its corresponding primer pairs [12]. The mixed RNA sample was used as template for reverse transcription to cDNA using MMLV reverse transcriptase (TOYOBO Co., Ltd., Osaka, Japan). The cDNA strand was then used as template in PCR to amplify a specific DNA band with the corresponding primer pairs listed in Additional file 1: Table S2, respectively.

### Phylogenetic analysis

Open reading frames (ORFs) and conserved domain(s) were determined by using the ORF Finder program and CD-search in NCBI. Then, the selected amino acid (aa) sequences of viral replicases or other conserved regions were used for the construction of phylogenetic trees with the neighbor-joining (NJ) method and tested with a bootstrap of 1000 replicates to ascertain the reliability of a given branch pattern in MEGA X software [17]. Multiple alignments of the sequences were performed using ClustalX.

## Results

### Viral sequences in the metatranscriptome of *Pestalotiopsis*

Fifty-nine *Pestalotiopsis* strains were isolated form the diseased samples collected from 14 locations (Additional file 1: Table S1). All these strains were determined to be *Pestalotiopsis* spp. based on our methods previously described [2, 3]. One sequencing library was constructed from the rRNA-depleted total RNAs of the 59 *Pestalotiopsis* strains. After the removal of all low-quality reads, 9.2 × 10^7^ reads with lengths > 20 nt (paired-end) were obtained, and a total of 164,628 contigs were assembled by using the software Trinity. A BLAST search of non-redundant protein database identified a total of 8 putative viral contigs (Table 1), possibly belonging to viral families *Botourmiaviridae*, *Narnaviridae*, *Deltaflexiviridae*, *Partitiviridae*, and the order *Bunyavirales*. The presence of these putative viral contigs in the *Pestalotiopsis* strains were confirmed by RT-PCR (Additional file 1: Fig. S1). Through conserved motif analysis, all contigs were found to possess a conserved domain of GDD except -ss viruses.

**Table 1:** Assembled sequences with similarity to previously described viruses

Contig number	GenBank ID	cDNA Contig length (bp)	Name of putative viruses	Best match^a^	aa identity (%)	Family/Genus
c61286_g1	MW017458	2486	Pestalotiopsis mitovirus 1	Sclerotinia homoeocarpa mitovirus (AAO21337.1)	49	*Mitoviridae*
c61448_g1	MW017459	2303	Pestalotiopsis mitovirus 2	Rhizoctonia solani mitovirus 10 (ALD89102.1)	44	*Mitoviridae*
c49783_g1	MT925636	2438	Colletotrichum gloeosporioides ourmia-like virus 1	Colletotrichum gloeosporioides ourmia-like virus 1 (QDW80875.1)	96	*Botourmiaviridae*
c55068_g1	MW017456	2674	Pestalotiopsis botourmiavirus 2	Plasmopara viticola associated ourmia-like virus 44 (QGY72574.1)	61	*Botourmiaviridae*
c59323_g1	MW017457	2460	Pestalotiopsis botourmiavirus 3	Plasmopara viticola associated ourmia-like virus 81 (QGY72611.1)	39	*Botourmiaviridae*
c43244_g1	MW017460	7667	Pestalotiopsis deltaflexi-like virus 1	Rhizoctonia solani flexivirus 1 (YP_009268715.1)	30	Unclassifified
c48689_g1	MT925635	7009	Pestalotiopsis negative-stranded RNA virus 1	Plasmopara associated mycobunyavirales-like virus 2 (QGY72639.1)	34	Unclassifified
c9266_g1	MT925634	1646	Pestalotiopsis partitivirus 1	Diatom colony associated dsRNA virus 14 (BAU79511.1)	49	*Partitiviridae*

^a^The Genbank accession number for each virus is listed in the parentheses

### Botourmiavirus-related sequences

**Fig. 1:**
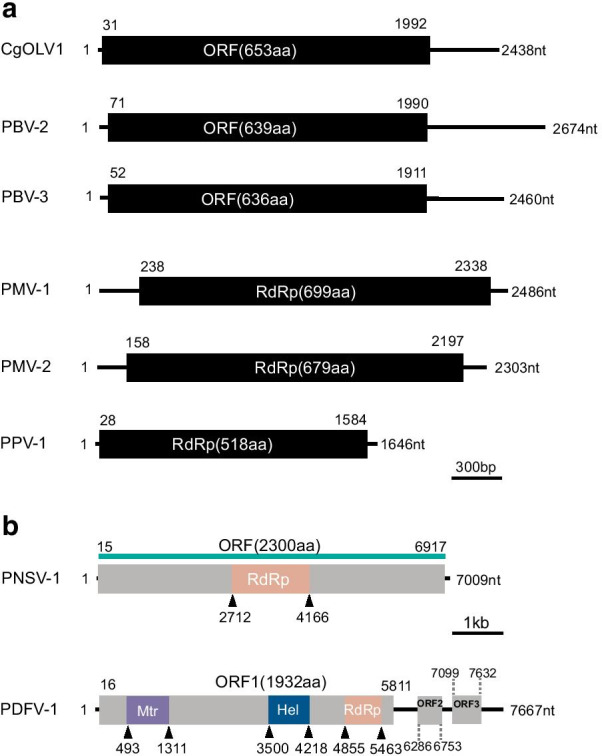
Schematic diagram showing the genome organization of viruses less than 3000 bp in length (**a**), including Colletotrichum gloeosporioides ourmia-like virus 1 (CgOLV1), Pestalotiopsis botourmiavirus 2 (PBV-2), Pestalotiopsis botourmiavirus 3 (PBV-3), Pestalotiopsis mitovirus 1 (PMV-1), Pestalotiopsis mitovirus 2 (PMV-2), and Pestalotiopsis partitivirus 1 (PPV-1); and viruses larger than 7000 bp in length (**b**), including Pestalotiopsis negative-stranded RNA virus 1 (PNSV-1) and Pestalotiopsis deltaflexi-like virus 1 (PDFV-1). The conserved domains include RNA dependent RNA polymerase (RdRp), methyltransferase (Mtr), and viral helicase (Hel) of PNSV-1 and PDFV-1, which are indicated on their genomes

**Fig. 2:**
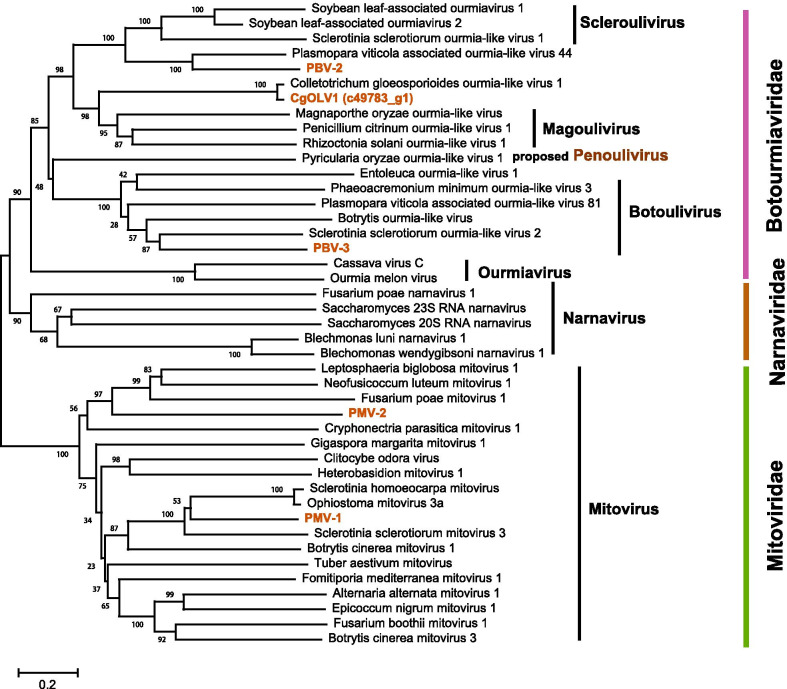
Phylogenetic analysis of Pestalotiopsis mitovirus 1, Pestalotiopsis mitovirus 2, Colletotrichum gloeosporioides ourmia-like virus 1 (c49783_g1), Pestalotiopsis botourmiavirus 2, and Pestalotiopsis botourmiavirus 3 based on the RNA dependent RNA polymerase. The GenBank accession number of each virus is listed in Additional file 1: Table S3. Numbers at the nodes are bootstrap values out of 1000 replicates

The fragment c49783_g1 was 2438 nt in length, possessing a large ORF encoding a 653 aa peptide showing homology to the RNA dependent RNA polymerase (RdRp) sequence of Colletotrichum gloeosporioides ourmia-like virus 1 (CgOLV1, QDW80875.1) with an aa sequence identity of 96%. Therefore, this fragment may represent a strain of CgOLV1. The fragment c55068_g1 was 2634 nt long, encoding a putative RdRp of 639 aa, which was 61% identical to the RdRp sequence of Plasmopara viticola associated ourmia-like virus 44 (QGY72574.1), and it was named Pestalotiopsis botourmiavirus 2 (PBV-2). The fragment c59323_g1 was 2460 nt in length encoding a putative RdRp of 636 aa, namely Pestalotiopsis botourmiavirus 3 (PBV-3). The RdRp of PBV-3 was most homologous to the RdRp sequence of Plasmopara viticola associated ourmia-like virus 81 (QGY72611.1) with aa sequence identity of 39% (Fig. 1a). Pairwise identity comparisons of PBV-2 and PBV-3 sequences to other known botoumiaviruses demonstrated that PBV-2 and PBV-3 shared < 68% genome identity with other known botoumiaviruses (Additional file 1: Fig. S2a). In addition, the multiple sequence alignments of the RdRps of CgOLV1, PBV-2, and PBV-3 detected eight conserved motifs that were characteristic of the RdRps of + ssRNA mycoviruses [18] (Additional file 1: Fig. S3). To establish the phylogeny of the three botoumiaviruses with other RNA viruses, a phylogenetic tree was constructed by using the NJ method based on the viral RdRp aa sequences. The results showed that PBV-3 was clustered with the botouliviruses. In contrast, PBV-2 was mostly related to Plasmopara viticola associated ourmia-like virus 44, likely belonging to the genus *Scleroulivirus*, while CgOLV1/c49783_g1 was mostly related to magouliviruses and might be a new member in this genus (Fig. 2).

### Mitovirus-related sequences

Two fragments, c61286_g1 (2486 nt) and c61286_g1 (2486 nt), were predicted to encode mitoviral RdRps, namely Pestalotiopsis mitovirus 1 (PMV-1) and Pestalotiopsis mitovirus 2 (PMV-2), respectively. The RdRp of PMV-1 was most similar to Sclerotinia homoeocarpa mitovirus (AAO21337.1), with an aa sequence identity of 49%, while PMV-2 was most similar to Rhizoctonia solani mitovirus 10 (ALD89102.1), with an aa sequence identity of 44% (Fig. 1a). Pairwise identity comparisons of PMV-1 and PMV-2 sequences to other known mitoviruses demonstrated that PMV-1 and PMV-2 shared < 69% genome identity with other known mitoviruses (Additional file 1: Fig. S2b). Multiple alignments based on viral RdRps indicated that RdRps of PMV-1 and PMV-2 contained six conserved motifs, which is the characteristic of mitoviruses [19] (Additional file 1: Fig. S4). Phylogenetic analysis using the RdRp aa sequences also showed that PMV-1 and PMV-2 were clustered with other mitoviruses based on the NJ method (Fig. 2).

### *Tymovirales*-related sequences

**Fig. 3:**
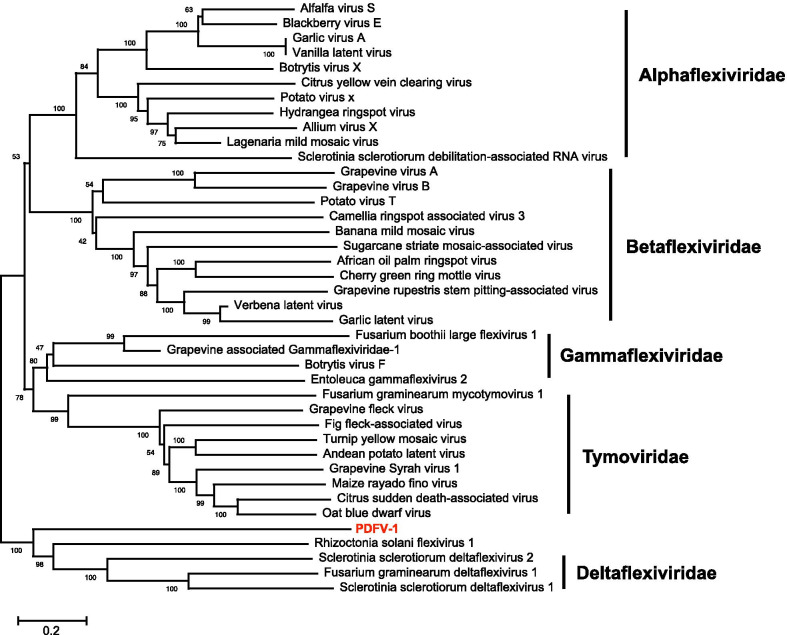
Phylogenetic analysis of Pestalotiopsis deltaflexi-like virus 1 based on the RNA dependent RNA polymerase. The GenBank accession number of each virus is listed in Additional file 1: Table S3. Numbers at the nodes are bootstrap values out of 1000 replicates

The fragment c43244_g1 was 7667 nt long and encoded a 1932 aa-long polyprotein, containing a methyltransferase (Mtr) and a viral helicase (Hel) domain (Fig. 1b). BLAST and phylogenetic analysis showed that the closest related virus to this fragment was Rhizoctonia solani flexivirus 1 (YP_009268715.1) with a sequence identity of 30% at the aa level. Therefore, it was temporarily named Pestalotiopsis deltaflexivirus 1 (PDFV-1). Furthermore, multiple alignments revealed that Mtr domain of PDFV-1 contained six conserved motifs, which were similar to the viral Mtr domains of Sclerotinia sclerotiorum deltaflexivirus 1 (NC_038977), Sclerotinia sclerotiorum deltaflexivirus 2 (NC_040649), and Fusarium graminearum deltaflexivirus 1 (NC_030654). In addition, the Hel domain of PDFV-1 was also similar to viruses in the family *Deltaflexiviridae*, and the RdRp domain of PDFV-1 contained six typical viral RdRp motifs similar to other deltaflexiviruses [35, 36] (Additional file 1: Fig. S5). To examine the phylogenetic relationship of PDFV-1 to viruses in *Tymovirales*, a phylogenetic tree was constructed based on the aa sequence of the polyproteins. The results showed that PDFV-1 was clustered with viruses of *Deltaflexiviridae* with 100% bootstrap support (Fig. 3).

### *Bunyavirales*-related sequences

**Fig. 4:**
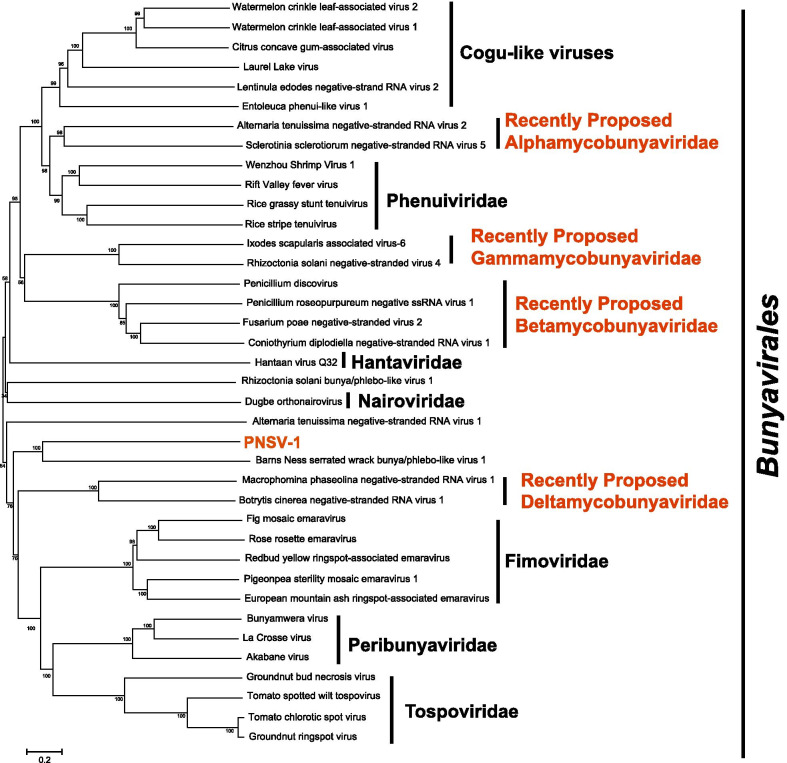
Phylogenetic analysis of Pestalotiopsis negative-stranded RNA virus 1 based on the RNA dependent RNA polymerase. The GenBank accession number of each virus is listed in Additional file 1: Table S3. Numbers at the nodes are bootstrap values out of 1000 replicates

In addition to + ssRNA viruses, a possible -ssRNA viral segment (c48689_g1) was also identified, namely Pestalotiopsis negative-stranded RNA virus 1 (PNSV-1). The identified PNSV-1 contig was 7009 nt long and putatively encoded a polyprotein of 2300 aa in length, containing an RdRp domain and a Hel domain (Fig. 1b). BLASTp analysis showed that the RdRp was 34% identical to that of Plasmopara associated mycobunyavirales-like virus 2 (QGY72639.1). The alignment of the RdRps of PfNSV-1, fig mosaic emaravirus, and rose rosette emaravirus indicated that the RdRps possessed five conserved motifs, which were also present in the RdRps of the typical members in *Bunyavirales* (Additional file 1: Fig. S6). Phylogenetic analysis revealed that PNSV-1 was grouped with Barns Ness serrated wrack bunya/phlebo-like virus 1 with 100% bootstrap support, which might belong to a novel viral family (Fig. 4).

### Partitivirus-related sequences

**Fig. 5:**
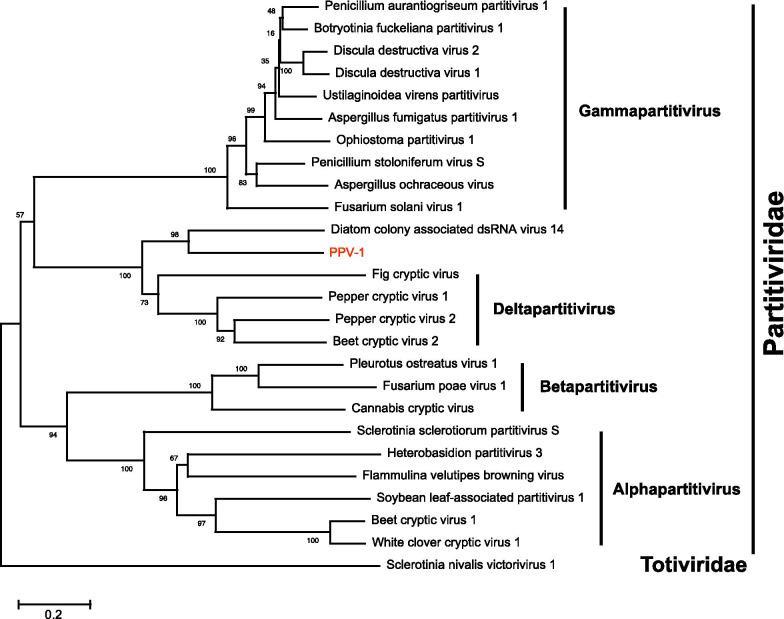
Phylogenetic analysis of Pestalotiopsis partitivirus 1 based on the RNA dependent RNA polymerase. The GenBank accession number of each virus is listed in Additional file 1: Table S3. Numbers at the nodes are bootstrap values out of 1000 replicates

According to the BLASTx search, only one fragment (c9266_g1) of 1646 bp in length, encoding a 518 aa long putative RdRp, showing homology to partitiviruses, namely Pestalotiopsis partitivirus 1 (PPV-1) (Fig. 1a). PPV-1 was most closely related to Diatom colony associated dsRNA virus 14, with the aa sequence identity of 49%. However, no coat protein (CP) sequence of PPV-1 was obtained. Eight conserved motifs were detected in PPV-1, as well as in beet cryptic virus 1, beet cryptic virus 2, beet cryptic virus 3, and epirus cherry virus, through multiple sequence alignments of the RdRps [20] (Additional file 1: Fig. S7). Phylogenetic analysis showed that PPV-1 was clustered with other deltapartitiviruses, forming an independent branch (Fig. 5).

## Discussion

Twig dieback of *M*. *rubra* caused by *Pestalotiopsis* spp. is a new threat to the production of *M*. *rubra* in China [2, 3]. The present study showed that various mycoviruses persist in the population of *Pestalotiopsis* spp. isolated from *M*. *rubra* in China. In this study, eight putative viral sequences were identified, many of which were nearly full-length. These viruses could be grouped into five distinct lineages, including *Botourmiaviridae*, *Narnaviridae*, *Partitiviridae*, *Tymovirales*, and *Bunyavirales*.

Viruses of the *Botourmiaviridae* usually possess a large open reading frame, encoding the viral replicase [21, 22], and there were three contigs that might belong to this family. In these viruses, two botourmiaviruses, PBV-2 and PBV-3, were novel and probably belonged to *Scleroulivirus* and *Botoulivirus*, respectively. It is of interest that the results showed that CgOLV1 was able to infect two different fungal species. Although the transmission of mycoviruses is generally believed to be restricted by vegetative incompatibility between the donor and receipt strains, viral interspecies transmission has been observed in some cases, such as those from *S*. *sclerotiorum* to *S*. *minor* [23], from *Cryphonectria parasitica* to *C*. *nitschkei* [24], and from *Aspergillus niger* to *A*. *nidulans* [25]. In addition, a few mycoviruses were reported to be present in different fungal hosts. For example, two mitoviruses, OnuMV3a and BcMV1 were detected in different fungal hosts, and BpBV1 may also be widely distributed in populations of *B. cinerea*, *B*. *squomosa*, and *S*. *sclerotiorum* [26, 27]. The -ssRNA virus, BcMyV1 was detected in populations of both *B*. *cinerea* and *S*. *sclerotiorum* [28]. Therefore, CgOLV1 may be able to be transmitted among different fungal groups. These data suggest that the transmission of mycoviruses among different fungal species may be widely occurring in natural conditions.

Mitoviruses are a group of naked viruses with linear + ssRNA genomes, possessing one large ORF that encodes an RdRp of 2.3–3.6 kb in size [29, 30], with few exceptions [31]. They are thought to replicate in mitochondria since they often use mitochondrial genetic code for the translation of RdRps [32, 33]. Two mitoviruses, PMV-1 and PMV-2, were identified in the present study, as both of them possessed the six characteristic conserved motifs of mitoviruses [19] (Additional file 1: Fig. S4). Phylogenetic analysis also supported this conclusion (Fig. 2). As both of them showed no sequence identity of > 90% to all reported mitoviruses, they should be two novel members in the genus *Mitovirus*.

The order *Tymovirales* is composed of five approved families (*Alpha*-, *Beta*-, *Delta*-, *Gammaflexiviridae*, and *Tymoviridae*) with viral genomes of + ssRNA, and all members contain a linear genome of 5.9–9.0 nt in length [34]. Only one possible member (PDFV-1) in this order was identified. Although the BLAST search showed that PDFV-1 was most closely related to Rhizoctonia solani flexivirus 1, the aa sequence identity between the two viruses was low at 30%. The conserved motifs in the Mtr, Hel, and RdRp domains of PDFV-1 were similar to those of deltaflexiviruses [35, 36]. Furthermore, phylogenetic analysis showed that PDFV-1 and Rhizoctonia solani flexivirus 1 were clustered with viruses in viral family *Deltaflexiviridae* with 100% bootstrap support. These results suggest that PDFV-1 may be a novel member of this family, but it is more distantly related to the reported deltaflexiviruses (Fig. 3).

Previous studies showed that most fungal -ss RNA viruses belonged to two orders, *Mononegavirales* and *Bunyavirales* [37]. Recent years, due to the development of metagenomics sequencing, diverse bunyaviruses have been reported in fungi, including *B. cinerea* [11], *Macrophomina phaseolina* [10], *Lentinula edodes* [38], *Penicillium roseopurpureum* [39], and some grapevine wood-inhabiting endophytic fungi [40]. In addition, bunyaviruses have also been detected in some oomycetes, such as *Pythium polare* [41] and *Halophytophthora* spp. [42]. A possible -ssRNA virus, PNSV-1, was identified in these Pestalotiopsis viruses. The phylogeny and conserved motif analysis indicated that PNSV-1 might belong to *Bunyavirales* but not *Mononegavirales*. PNSV-1 was firstly grouped with barns ness serrated wrack bunya/phlebo-like virus 1 with 100% bootstrap support (Fig. 4), without showing high sequence similarity with other bunyaviruses. Therefore, PNSV-1 cannot be classified into any reported viral family and should belong to a new viral family within the order *Bunyavirales*.

Partitiviruses are a widely spread group of viruses infecting plants, fungi, and protozoa. Most partitiviruses possess two dsRNA genomes, of which one encodes the RdRp, while the other encodes the CP [43]. The family *Partitiviridae* contains five genera, *Alphapartitivirus*, *Betapartitivirus*, *Gammapartitivirus*, *Deltapartitivirus*, and *Cryspovirus*, of which alphapartitiviruses and betapartitiviruses infect both plants and fungi, whereas gammapartitiviruses and deltapartitiviruses infect only fungi and plants [43]. PPV-1 was the only identified partitivirus in the present study. Its RdRp was most closely related to diatom colony associated dsRNA virus 14 (DcV14) with a low sequence identity of 49%. However, no CP sequence of PPV-1 was obtained. Phylogenetic analysis showed PPV-1 was clustered with other deltapartitiviruses with 100% bootstrap support. However, PPV-1 formed an independent branch with DcV14 in the deltapartitivirus clade, indicating that it is distantly related to the present members in this genus (Fig. 5). As the reported deltapartitiviruses only infect plants, this result indicates that deltapartitiviruses may not only infect plants but also fungi.

This is the first report showing the existence of various mycoviruses in *Pestalotiopsis* spp. isolated from *M*. *rubra* in China. In this study, we identified sequences that putatively represented the genomes of 8 viruses, many of which were nearly full-length. However, the effects of these viruses on *Pestalotiopsis* spp. remain unknown and needed to be investigated in further studies. Accessing virus mediated effects on *Pestalotiopsis* spp. will be helpful for further development of these viruses as biocontrol agents for the control of twig dieback in *M*. *rubra*.

## Conclusions

This is the first study showing the existence of various mycoviruses in *Pestalotiopsis* spp. isolated from *M*. *rubra* in China. In this study, eight mycoviruses were identified and could be assigned into five distinct lineages, including *Botourmiaviridae*, *Narnaviridae*, *Partitiviridae*, *Tymovirales*, and *Bunyavirales*. Within these viruses, PNSV-1 may belong to a novel viral family. Through metetranscriptomic analysis, more mycoviral groups were detected and may be tapped as biocontrol agents for fungal diseases of plants.

## Supplementary Information

**Additional file 1**. **Figure S1**. RT-PCR confirmation of eight viral-like contigs in the mixed RNA sample. See Table S2 for detailed information of primers and amplicon sizes. **Figure S2**. Pairwise identity comparisons of botoumiaviral (a) and mitoviral (b) sequences in *Pestalotiopsis* strains to other known botoumiaviruses and mitoviruses. See Table S3 for detailed information of each virus. **Figure S3**. Conserved amino acid sequence motifs of the putative RNA-dependent RNA polymerases of Colletotrichum gloeosporioides ourmia-like virus 1 (CgOLV1), Pestalotiopsis botourmiavirus 2 (PBV-2), Pestalotiopsis botourmiavirus 3 (PBV-3), Botrytis ourmia-like virus (BOLV), Phoma matteucciicola ourmia-like virus 1 (PmOLV1), Magnaporthe oryzae ourmia-like virus (MOLV), and Rhizoctonia solani ourmia-like virus 1 (RsOLV1). The GenBank accession number of each virus is listed in Table S3. “*” indicates identical amino acid residues; and “.” indicates low chemically similar amino acid residues. **Figure S4**. Conserved amino acid sequence motifs of the putative RNA-dependent RNA polymerases of Pestalotiopsis mitovirus 1 (PMV-1), Pestalotiopsis mitovirus 2 (PMV-2), Cryphonectria cubensis mitovirus 2a (CcMV2a), Ophiostoma mitovirus 1a (OnuMV1a), Sclerotinia homoeocarpa mitovirus (ShMV), Ophiostoma mitovirus 3a (OnuMV1a), and Cryphonectria parasitica mitovirus 1-NB631 (CpMV1-NB631). The GenBank accession number of each virus is listed in Table S3. “*” indicates identical amino acid residues; and “.” indicates low chemically similar amino acid residues. **Figure S5**. Multiple alignment of amino acid sequences of conserved domains including methyltransferase (Mtr), viral RNA Helicase (Hel), and RNA-dependent RNA polymerase (RdRp) domains of Pestalotiopsis deltaflexi-like virus 1 (PDFV-1), Sclerotinia sclerotiorum deltaflexivirus 1 (SsDFV1), Sclerotinia sclerotiorum deltaflexivirus 2 (SsDFV2), and Fusarium graminearum deltaflexivirus 1 (FgDFV1). The GenBank accession number of each virus is listed in Table S3. “*” indicates identical amino acid residues; and “.” indicates low chemically similar amino acid residues. **Figure S6**. Conserved amino acid sequence motifs of the putative RNA-dependent RNA polymerases of Pestalotiopsis negative-stranded RNA virus 1, rose rosette emaravirus (RRV), and fig mosaic emaravirus (FMV). The GenBank accession number of each virus is listed in Table S3. “*” indicates identical amino acid residues; and “.” indicates low chemically similar amino acid residues. **Figure S7**. Conserved amino acid sequence motifs of the putative RNA-dependent RNA polymerases of Pestalotiopsis partitivirus 1 (PPV-1), beet cryptic virus 1 (BcV1), beet cryptic virus 2 (BcV2), beet cryptic virus 3 (BcV3), and fig cryptic virus (FcV). The GenBank accession number of each virus is listed in Table S3. “*” indicates identical amino acid residues; and “.” indicates low chemically similar amino acid residues. **Table S1**. Strains used in this study. **Table S2**. List of PCR primers used for viral contig detection. **Table S3**. Viruses used for multiple sequence alignments and phylogenetic analysis.

## Data Availability

The viral sequences are available on NCBI at https://www.ncbi.nlm.nih.gov/. The raw data used for metetranscriptomic analysis are available at the Bioproject PRJNA661377 in NCBI.

## Abbreviations

NGS: Next-generation sequencing
PDA: Potato dextrose agar
NCBI: National Center for Biotechnology Information
ORF: Open reading frame
NJ: Neighbor-joining
RdRp: RNA dependent RNA polymerase
CgOLV1: Colletotrichum gloeosporioides ourmia-like virus 1
PBV-2: Pestalotiopsis botourmiavirus 2
PMV-1: Pestalotiopsis mitovirus 1
PMV-2: Pestalotiopsis mitovirus 2
PDFV-1: Pestalotiopsis deltaflexivirus 1
PNSV-1: Pestalotiopsis negative-stranded RNA virus 1
PPV-1: Pestalotiopsis partitivirus 1
DcV14: Diatom colony associated dsRNA virus 14
