# Supplementary material for: Multiple mycoviruses identified in Pestalotiopsis spp. from Chinese bayberry
Source: Virol J. 2021 Feb 23;18:43. doi: 10.1186/s12985-021-01513-3 (PMC7903649; doi:10.1186/s12985-021-01513-3)
Supplement: Supplementary file 1 — Additional file 1. Figure S1. RT-PCR confirmation of eight viral-like contigs in the mixed RNA sample. See Table S2 for detailed information of primers and amplicon sizes. Figure S2. Pairwise identity comparisons of botoumiaviral (a) and mitoviral (b) sequences in Pestalotiopsis strains to other known botoumiaviruses and mitoviruses. See Table S3 for detailed information of each virus. Figure S3. Conserved amino acid sequence motifs of the putative RNA-dependent RNA polymerases of Colletotrichum gloeosporioides ourmia-like virus 1 (CgOLV1), Pestalotiopsis botourmiavirus 2 (PBV-2), Pestalotiopsis botourmiavirus 3 (PBV-3), Botrytis ourmia-like virus (BOLV), Phoma matteucciicola ourmia-like virus 1 (PmOLV1), Magnaporthe oryzae ourmia-like virus (MOLV), and Rhizoctonia solani ourmia-like virus 1 (RsOLV1). The GenBank accession number of each virus is listed in Table S3. “*” indicates identical amino acid residues; and “.” indicates low chemically similar amino acid residues. Figure S4. Conserved amino acid sequence motifs of the putative RNA-dependent RNA polymerases of Pestalotiopsis mitovirus 1 (PMV-1), Pestalotiopsis mitovirus 2 (PMV-2), Cryphonectria cubensis mitovirus 2a (CcMV2a), Ophiostoma mitovirus 1a (OnuMV1a), Sclerotinia homoeocarpa mitovirus (ShMV), Ophiostoma mitovirus 3a (OnuMV1a), and Cryphonectria parasitica mitovirus 1-NB631 (CpMV1-NB631). The GenBank accession number of each virus is listed in Table S3. “*” indicates identical amino acid residues; and “.” indicates low chemically similar amino acid residues. Figure S5. Multiple alignment of amino acid sequences of conserved domains including methyltransferase (Mtr), viral RNA Helicase (Hel), and RNA-dependent RNA polymerase (RdRp) domains of Pestalotiopsis deltaflexi-like virus 1 (PDFV-1), Sclerotinia sclerotiorum deltaflexivirus 1 (SsDFV1), Sclerotinia sclerotiorum deltaflexivirus 2 (SsDFV2), and Fusarium graminearum deltaflexivirus 1 (FgDFV1). The GenBank accession number of each virus is listed in T [file 12985_2021_1513_MOESM1_ESM.pdf]

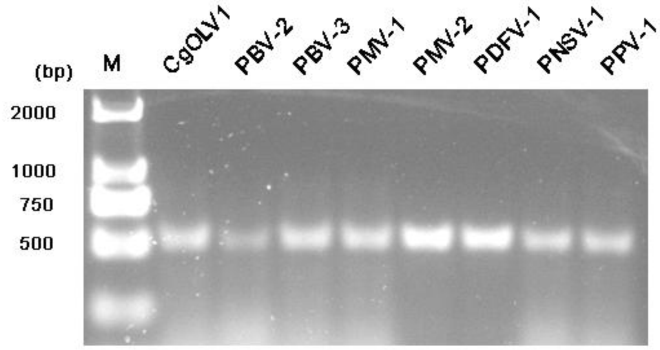

Figure S1. RT-PCR confirmation of eight viral-like contigs in the mixed RNA sample. See Table S2 for detailed information of primers and amplicon sizes.

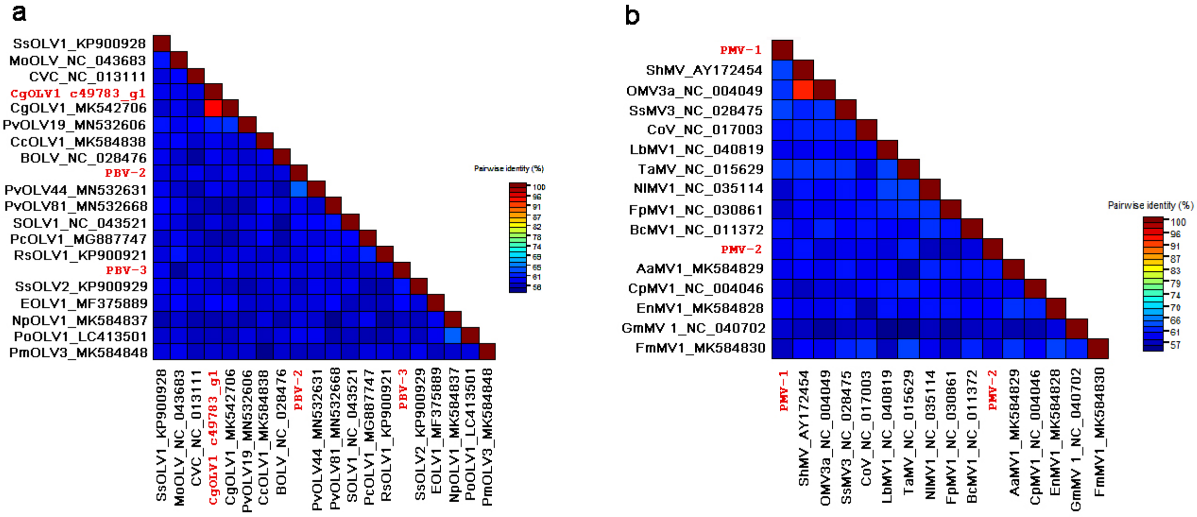

Figure S2. Pairwise identity comparisons of botoumiaviral (a) and mitoviral (b) sequences in *Pestalotiopsis* strains to other known botoumiaviruses and mitoviruses. See Table S3 for detailed information of each virus.

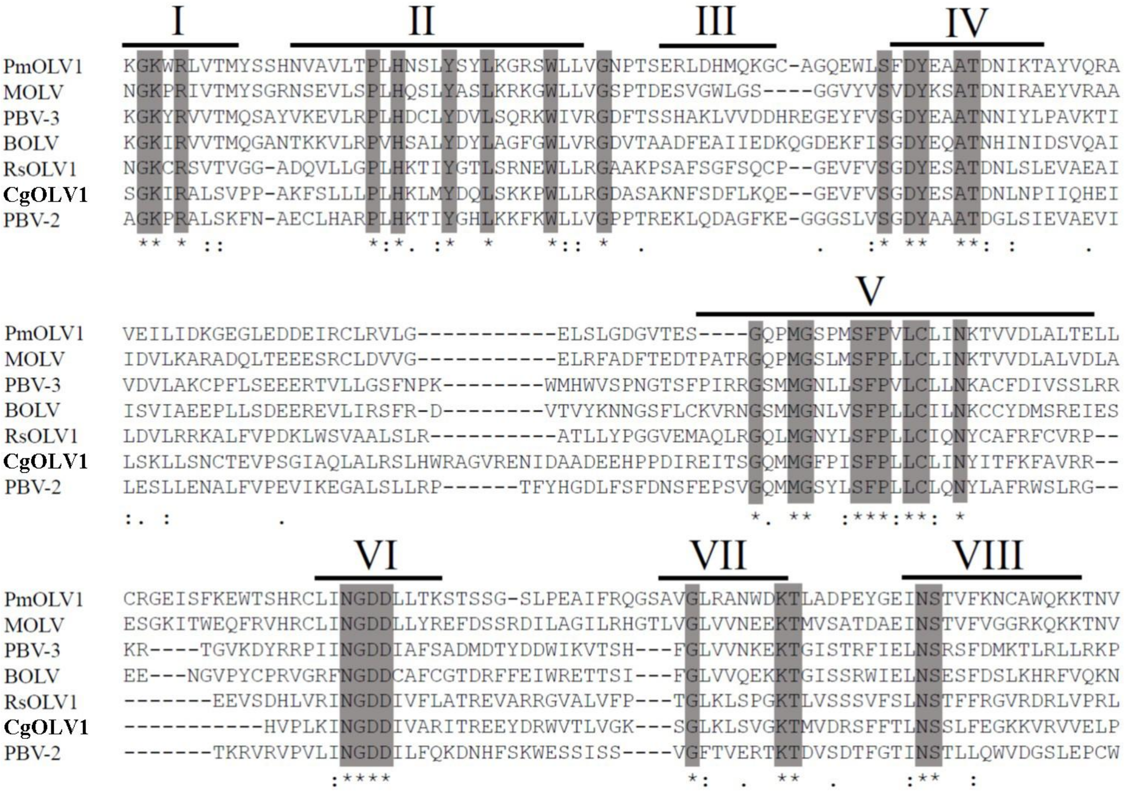

Figure S3. Conserved amino acid sequence motifs of the putative RNA-dependent RNA polymerases of Colletotrichum gloeosporioides ourmia-like virus 1 (CgOLV1/c49783_g1), Pestalotiopsis botourmiavirus 2 (PBV-2), Pestalotiopsis botourmiavirus 3 (PBV-3), Botrytis ourmia-like virus (BOLV), Phoma matteucciicola ourmia-like virus 1 (PmOLV1), Magnaporthe oryzae ourmia-like virus (MOLV), and Rhizoctonia solani ourmia-like virus 1 (RsOLV1). The GenBank accession number of each virus is listed in Table S3. "*" indicates identical amino acid residues; and "." indicates low chemically similar amino acid residues.

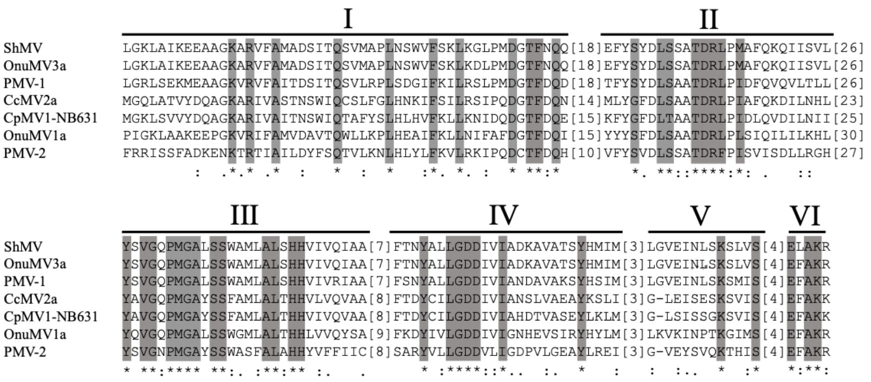

Figure S4. Conserved amino acid sequence motifs of the putative RNA-dependent RNA polymerases of Pestalotiopsis mitovirus 1 (PMV-1), Pestalotiopsis mitovirus 2 (PMV-2), Cryphonectria cubensis mitovirus 2a (CcMV2a), Ophiostoma mitovirus 1a (OnuMV1a), Sclerotinia homoeocarpa mitovirus (ShMV), Ophiostoma mitovirus 3a (OnuMV1a), and Cryphonectria parasitica mitovirus 1-NB631 (CpMV1-NB631). The GenBank accession number of each virus is listed in Table S3. "*" indicates identical amino acid residues; and "." indicates low chemically similar amino acid residues.

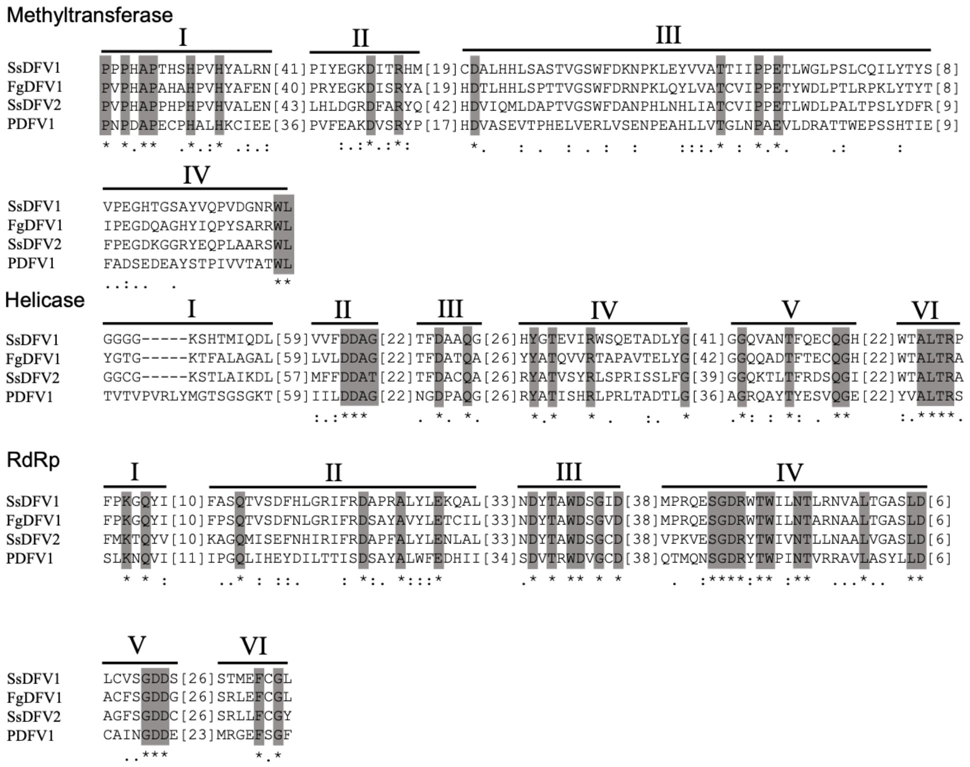

Figure S5. Multiple alignment of amino acid sequences of conserved domains including methyltransferase (Mtr), viral RNA Helicase (Hel), and RNA-dependent RNA polymerase (RdRp) domains of Pestalotiopsis deltaflexi-like virus 1 (PDFV-1), Sclerotinia sclerotiorum deltaflexivirus 1 (SsDFV1), Sclerotinia sclerotiorum deltaflexivirus 2 (SsDFV2), and Fusarium graminearum deltaflexivirus 1 (FgDFV1). The GenBank accession number of each virus is listed in Table S3. "*" indicates identical amino acid residues; and "." indicates low chemically similar amino acid residues.

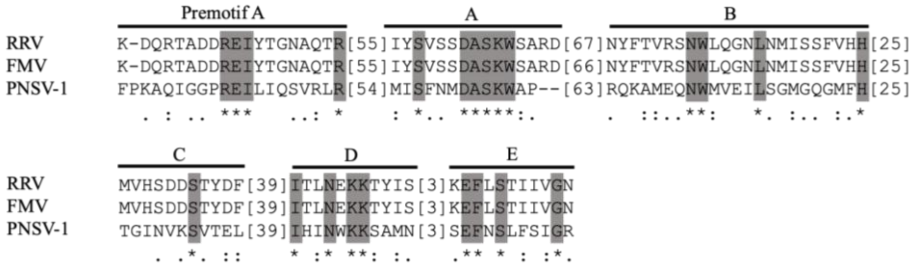

Figure S6. Conserved amino acid sequence motifs of the putative RNA-dependent RNA polymerases of Pestalotiopsis negative-stranded RNA virus 1, rose rosette emaravirus (RRV), and fig mosaic emaravirus (FMV). The GenBank accession number of each virus is listed in Table S3. "*" indicates identical amino acid residues; and "." indicates low chemically similar amino acid residues.

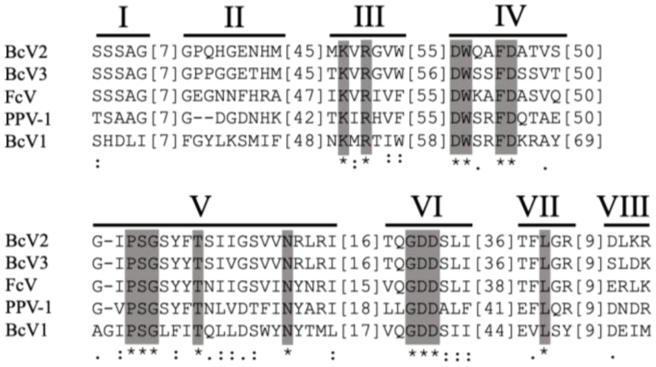

Figure S7. Conserved amino acid sequence motifs of the putative RNA-dependent RNA polymerases of Pestalotiopsis partitivirus 1 (PPV-1), beet cryptic virus 1 (BcV1), beet cryptic virus 2 (BcV2), beet cryptic virus 3 (BcV3), and fig cryptic virus (FcV). The GenBank accession number of each virus is listed in Table S3. “*” indicates identical amino acid residues; and “.” indicates low chemically similar amino acid residues.

Table S1. Strains used in this study

Sample	Location	Strain
TD-1, TD-2, TD-3	Luqiao, Zhejiang	CBP-1, CBP-2, CBP-3, CBP-4
TD-4, TD-5	Lanxi, Zhejiang	CBP-5, CBP-6, CBP-7, CBP-8
TD-6, TD-7	Yueqing, Zhejiang	CBP-9, CBP-10, CBP-11, CBP-12
TD-8, TD-9, TD-10	Taishun, Zhejiang	CBP-13, CBP-14, CBP-15, CBP-16
TD-11, TD-12	Xiangshan, Zhejiang	CBP-17, CBP-18, CBP-19, CBP-20
TD-13, TD-14, TD-15	Wenling, Zhejiang	CBP-21, CBP-22, CBP-23, CBP-24, CBP-25
TD-16, TD-17	Linghai, Zhejiang	CBP-26, CBP-27, CBP-28, CBP-29
TD-18, TD-19, TD-20	Huangyan, Zhejiang	CBP-30, CBP-31, CBP-32, CBP-33
TD-21, TD-22, TD-23	Lishui, Zhejiang	CBP-34, CBP-35, CBP-36, CBP-37
TD-24, TD-25, TD-26	Yubei, Chongqing	CBP-38, CBP-39, CBP-40, CBP-41, CBP-42
TD-27, TD-28, TD-29	Fuan, Fujiang	CBP-43, CBP-44, CBP-45, CBP-46
TD-30, TD-31	Ruili, Yunnan	CBP-47, CBP-48, CBP-49, CBP-50
TD-32, TD-33	Yingtan, Jiangxi	CBP-51, CBP-52, CBP-53, CBP-54
TD-34, TD-35	Leshan, Sichuan	CBP-55, CBP-56, CBP-57, CBP-58, CBP-59

Table S2. List of PCR primers used for viral contig detection

Virus	Primer Forward(5′-3′)	Primer Reverse(5′-3′)	Product Size (bp)
PPV-1	CACTCCATAACTATGGCTCGGAC	GAACATGTCTTTTCCGGTGAGG	521
PBV-1	TCTCTAAACTTCTCAGCAATTGTA	TCTTCTGTCCAAAGTATCCG	531
PBV-2	CGAGTGTTTGCACGCTCGG	CTTAGTCCGCTCTACCGTAAAG	526
PBV-3	GTGGAGAGGGAGGGACACTA	CGAAGGGAACTCACAATGTCG	523
PMV-1	CGTTTATTAGGCCTTCATAAAG	GGAATACCTTTTAAAGTTTTAAG	536
PMV-2	ACCTGGAATCTATAACCGGAC	TAAGTCAACACTATAGAATACCGG	530
PDFV1	CGGGAGCATCCGATCCCAA	GTGGAGATAGCGTTGTACAAAA	521
PNSV-1	GGTGGAGGTGGAGGCGGT	AGTCGACGTCGAACTCCG	512

Table S3. Viruses used for multiple sequence alignments and phylogenetic analysis

Name	GenBank accession number	Family
Soybean leaf-associated ourmiavirus 1	NC_043521	Botourmiaviridae
Soybean leaf-associated ourmiavirus 2	NC_043522	Botourmiaviridae
Sclerotinia sclerotiorum ourmia-like virus 1	KP900928	Botourmiaviridae
Plasmopara viticola associated ourmia-like virus 44	MN532631	Botourmiaviridae
Colletotrichum gloeosporioides ourmia-like virus 1	MK542706	Botourmiaviridae
Magnaporthe oryzae ourmia-like virus	NC_043683	Botourmiaviridae
Penicillium citrinum ourmia-like virus 1	MG887747	Botourmiaviridae
Rhizoctonia solani ourmia-like virus 1	KP900922.1	Botourmiaviridae
Pyricularia oryzae ourmia-like virus 1	LC413501	Botourmiaviridae
Entoleuca ourmia-like virus 1	MF375889	Botourmiaviridae
Phaeoacremonium minimum ourmia-like virus 3	MK584848	Botourmiaviridae
Plasmopara viticola associated ourmia-like virus 81	MN532668	Botourmiaviridae
Phoma matteucciicola ourmia-like virus 1	QIP68359	Botourmiaviridae
Rhizoctonia solani ourmia-like virus 1	ALD89131	Botourmiaviridae
Botrytis ourmia-like virus	MT119674	Botourmiaviridae
Sclerotinia sclerotiorum ourmia-like virus 2	KP900929	Botourmiaviridae
Cassava virus C	NC_013113	Botourmiaviridae
Ourmia melon virus	NC_011070	Botourmiaviridae
Fusarium poae narnavirus 1	NC_030865	Narnaviridae
Saccharomyces 23S RNA narnavirus	NC_004050	Narnaviridae
Saccharomyces 20S RNA narnavirus	NC_004051	Narnaviridae
Blechmonas luni narnavirus 1	NC_040829	Narnaviridae
Blechomonas wendygibsoni narnavirus 1	NC_040641	Narnaviridae
Leptosphaeria biglobosa mitovirus 1	NC_040819	Mitoviridae
Neofusicoccum luteum mitovirus 1	NC_035114	Mitoviridae
Fusarium poae mitovirus 1	NC_030861	Mitoviridae
Cryphonectria parasitica mitovirus 1	NC_004046	Mitoviridae
Cryphonectria parasitica mitovirus 1-NB631	NP_660174	Mitoviridae
Ophiostoma mitovirus 1a	CAJ32466	Mitoviridae
Cryphonectria cubensis mitovirus 2a	AAR01973	Mitoviridae
Gigaspora margarita mitovirus 1	NC_040702	Mitoviridae
Clitocybe odora virus	NC_017003	Mitoviridae
Heterobasidion mitovirus 1	KJ873059	Mitoviridae
Sclerotinia homoeocarpa mitovirus	AY172454	Mitoviridae
Ophiostoma mitovirus 3a	NC_004049	Mitoviridae
Sclerotinia sclerotiorum mitovirus 3	NC_028475	Mitoviridae
Botrytis cinerea mitovirus 1	NC_011372	Mitoviridae
Tuber aestivum mitovirus	NC_015629	Mitoviridae
Fomitiporia mediterranea mitovirus 1	MK584830	Mitoviridae
Alternaria alternata mitovirus 1	MK584829	Mitoviridae
Epicoccum nigrum mitovirus 1	MK584828	Mitoviridae

Fusarium boothii mitovirus 1	LC425114	Mitoviridae
Botrytis cinerea mitovirus 3	NC_028472	Mitoviridae
Alfalfa virus S	NC_034622	Alphaflexiviridae
Blackberry virus E	NC_015706	Alphaflexiviridae
Garlic virus A	NC_003375	Alphaflexiviridae
Vanilla latent virus	NC_035204	Alphaflexiviridae
Botrytis virus X	NC_005132	Alphaflexiviridae
Citrus yellow vein clearing virus	NC_026592	Alphaflexiviridae
Potato virus x	NC_011620	Alphaflexiviridae
Hydrangea ringspot virus	NC_006943	Alphaflexiviridae
Allium virus X	NC_012211	Alphaflexiviridae
Lagenaria mild mosaic virus	NC_043079	Alphaflexiviridae
Sclerotinia sclerotiorum debilitation-associated RNA virus	NC_007415	Alphaflexiviridae
Grapevine virus A	NC_003604	Betaflexiviridae
Grapevine virus B	NC_003602	Betaflexiviridae
Potato virus T	NC_011062	Betaflexiviridae
Camellia ringspot associated virus 3	MK050796	Betaflexiviridae
Banana mild mosaic virus	NC_002729	Betaflexiviridae
Sugarcane striate mosaic-associated virus	NC_003870	Betaflexiviridae
African oil palm ringspot virus	NC_012519	Betaflexiviridae
Cherry green ring mottle virus	NC_001946	Betaflexiviridae
Grapevine rupestris stem pitting-associated virus	NC_001948	Betaflexiviridae
Verbena latent virus	NC_043085	Betaflexiviridae
Garlic latent virus	NC_003557	Betaflexiviridae
Fusarium boothii large flexivirus 1	LC425116	Gammaflexiviridae
Grapevine associated Gammaflexiviridae-1	HM852917	Gammaflexiviridae
Botrytis virus F	NC_002604	Gammaflexiviridae
Entoleuca gammaflexivirus 2	MF375884	Gammaflexiviridae
Fusarium graminearum mycotymovirus 1	NC_040784	Tymoviridae
Grapevine fleck virus	NC_003347	Tymoviridae
Fig fleck-associated virus	NC_015229	Tymoviridae
Turnip yellow mosaic virus	NC_004063	Tymoviridae
Andean potato latent virus	NC_020470	Tymoviridae
Grapevine Syrah virus 1	NC_012484	Tymoviridae
Maize rayado fino virus	NC_002786	Tymoviridae
Citrus sudden death-associated virus	NC_006950	Tymoviridae
Oat blue dwarf virus	NC_001793	Tymoviridae
Rhizoctonia solani flexivirus 1	NC_030655	unclassified
Sclerotinia sclerotiorum deltaflexivirus 2	NC_040649	Deltaflexiviridae
Fusarium graminearum deltaflexivirus 1	NC_030654	Deltaflexiviridae
Sclerotinia sclerotiorum deltaflexivirus 1	NC_038977	Deltaflexiviridae
Fusarium graminearum negative-stranded RNA virus 1	MF276904	Mymonaviridae

Soybean leaf-associated negative-stranded RNA virus 1	KT598225	Mymonaviridae
Alternaria tenuissima negative-stranded RNA virus 1	MK584852	Mymonaviridae
Soybean leaf-associated negative-stranded RNA virus 2	KT598227	Mymonaviridae
Rose rosette emaravirus	YP_004327589	Mymonaviridae
Fig mosaic emaravirus	YP_009237269	Mymonaviridae
sderotinia sclerotiorum negative-stranded RNA virus 1	NC_025383	Mymonaviridae
sderotinia sclerotiorum negative-stranded RNA virus 3	NC_026732	Mymonaviridae
Botrytis cinereal mymonavirus 1	MH648611	Mymonaviridae
Kiln barn virus	MF893248	Mymonaviridae
Penicillium adametzioides negative-stranded RNA virus 1	MK584858	Mymonaviridae
Soybean leaf-associated negative-stranded RNA virus 4	KT598229	Mymonaviridae
Soybean leaf-associated negative-stranded RNA virus 3	KT598228	Mymonaviridae
Sclerotinia sclerotiorum negative-stranded RNA virus 2	KP900931	Mymonaviridae
Sclerotinia sclerotiorum negative-stranded RNA virus 4	NC_043483	Mymonaviridae
Barley yellow striate mosaic cytorhabdovirus	NC_028244	Rhabdoviridae
Northern cereal mosaic cytorhabdovirus	NC_002251	Rhabdoviridae
Maize fine streak nucleorhabdovirus	NC_005974	Rhabdoviridae
Eggplant mottled dwarf nucleorhabdovirus	NC_025389	Rhabdoviridae
Potato yellow dwarf nucleorhabdovirus	NC_016136	Rhabdoviridae
Mumps rubulavirus	NC_002200	Paramyxoviridae
Nipah henipavirus	NC_002728	Paramyxoviridae
Canine morbillivirus	NC_001921	Paramyxoviridae
Measles morbillivirus	NC_001498	Paramyxoviridae
Lettuce ring necrosis virus	NC_006054	Aspiviridae
Ranunculus white mottle virus	NC_043389	Aspiviridae
Blueberry mosaic associated virus	NC_036635	Aspiviridae
Cladosporium cladosporioides negative-stranded RNA virus 1	MK584856	unclassified
Fusarium poae negative-stranded virus 1	NC_030871	unclassified
Rhizoctonia solani negative-stranded virus 1	KP900919	unclassified
Rhizoctonia solani negative-stranded virus 2	KP900920	unclassified
Rhizoctonia solani negative-stranded virus 3	KP900903	unclassified
Botrytis cinerea negative-stranded RNA virus 1	NC_028466	unclassified
Macrophomina phaseolina negative-stranded RNA virus	KP900899	unclassified

Rice grassy stunt tenuivirus	NC_002328	Phenuiviridae
Rice stripe tenuivirus	NC_003776	Phenuiviridae
Rift valley fever virus	NC_014395	Phenuiviridae
Wenzhou Shrimp Virus 1	NC_031292	Phenuiviridae
Alternaria tenuissima negative-stranded RNA virus 2 AtNsRV2	MK584855	unclassified
Sclerotinia sclerotiorum negative-stranded RNA virus 5	MF444283	unclassified
Coniothyrium diplodiella negative-stranded RNA virus 1	MN532680	unclassified
Rhizoctonia solani negative-stranded virus 4	KP900923	unclassified
Cladosporium cladosporioides negative-stranded RNA virus 2 CcNsRV2	MK584857	unclassified
Hantaan virus	NC_005222	Hantaviridae
Groundnut ringspot virus	NC_043503	Tospoviridae
Tomato chlorotic spot virus	NC_035484	Tospoviridae
Tomato spotted wilt tospovirus	NC_002052	Tospoviridae
Groundnut bud necrosis virus	MK875278	Tospoviridae
La Crosse virus	NC_004110	Peribunyaviridae
Bunyamwera virus	NC_001927	Peribunyaviridae
Akabane virus	NC_009895	Peribunyaviridae
Rose rosette emaravirus	NC_034981	Fimoviridae
Fig mosaic emaravirus	NC_029568	Fimoviridae
Redbud yellow ringspot-associated emaravirus	NC_038856	Fimoviridae
European mountain ash ringspot-associated emaravirus	NC_013105	Fimoviridae
Penicillium aurantiogriseum partitivirus 1	NC_028499	Gammapartitivirus
Botryotinia fuckeliana partitivirus 1	NC_010350	Gammapartitivirus
Discula destructiva virus 2	NC_003711	Gammapartitivirus
Discula destructiva virus 1	NC_002800	Gammapartitivirus
Ustilaginoidea virens partitivirus	KJ868799	Gammapartitivirus
Aspergillus fumigatus partitivirus 1	LR746165	Gammapartitivirus
Ophiostoma partitivirus 1	NC_038918	Gammapartitivirus
Penicillium stoloniferum virus S	NC_005977	Gammapartitivirus
Aspergillus ochraceous virus	NC_043397	Gammapartitivirus
Fusarium solani virus 1	NC_003886	Gammapartitivirus
Diatom colony associated dsRNA virus 14	AP014907	unclassified
Fig cryptic virus	YP_004429258	Deltapartitivirus
Pepper cryptic virus 1	NC_037096	Deltapartitivirus
Pepper cryptic virus 2	NC_034159	Deltapartitivirus
Beet cryptic virus 2	YP_009508068	Deltapartitivirus
Beet cryptic virus 1	YP_002308574	Deltapartitivirus
Beet cryptic virus 3	YP_009665971	Deltapartitivirus
Pleurotus ostreatus virus 1	NC_006961	Betapartitivirus

Fusarium poae virus 1	NC_003884	Betapartitivirus
Cannabis cryptic virus	NC_031134	Betapartitivirus
Sclerotinia sclerotiorum partitivirus S	NC_013015	Alphapartitivirus
Heterobasidion partitivirus 3	NC_038836	Alphapartitivirus
Flammulina velutipes browning virus	NC_038826	Alphapartitivirus
Soybean leaf-associated partitivirus 1	KT598242	Alphapartitivirus
Epirus cherry virus	YP_002019754	Alphapartitivirus
White clover cryptic virus 1	NC_006276	Alphapartitivirus
Sclerotinia nivalis victorivirus 1	NC_030392	Totiviridae
